# Cochlear Implants Versus Hearing Aids in Children With Auditory Neuropathy Spectrum Disorder: A Systematic Review

**DOI:** 10.7759/cureus.117160

**Published:** 2026-09-29

**Authors:** Ali I Swaid, Reham T Alruwaili, Mazen A Alayidh, Abdulmajeed M Alwzynani, Hussain M Reda, Hassan H AlAli, Atallah A Almadi, Ali A Alshehri, Yasser H Alatawi, Raed H Aljabri, Refal Morea

**Affiliations:** 1 Faculty of Medicine, Jazan University, Jazan, SAU; 2 General Practice, Jouf University, Aljouf, SAU; 3 College of Medicine, King Khalid University, Abha, SAU; 4 College of Medicine, Umm AlQura University, Makkah, SAU; 5 Faculty of Medicine, Dar Al Uloom University, Riyadh, SAU; 6 Faculty of Medicine, Tanta University, Tanta, EGY; 7 Faculty of Medicine, Tabuk University, Tabuk, SAU; 8 Faculty of Medicine, Batterjee Medical Collage, Jeddah, SAU

**Keywords:** amplification, auditory neuropathy spectrum disorder, auditory outcomes, children, cochlear implantation, communication outcomes, hearing aids, pediatric hearing loss, speech perception, systematic review

## Abstract

Auditory neuropathy spectrum disorder (ANSD) is a heterogeneous hearing disorder characterized by impaired auditory neural synchrony and variable auditory and communication outcomes. Hearing aids (HAs) are commonly used as initial treatment, whereas cochlear implantation (CI) may be considered for children who show inadequate progress with amplification. This systematic review compared auditory and communication outcomes of CI and HAs in children with ANSD. PubMed/MEDLINE, Web of Science, Scopus, and the Cochrane Central Register of Controlled Trials were searched through 6 August 2026 for comparative studies reporting quantitative auditory or communication outcomes for both CI and HA groups. Because outcomes were reported using heterogeneous measures and formats, findings were synthesized narratively, with one dichotomous outcome analyzed quantitatively. Open-set speech perception outcomes showed substantial overlap between children using CI and HAs, and no study demonstrated a statistically significant difference between devices. In one study, at least 60% open-set speech perception was achieved by 6/16 (37.5%) children with CI and 4/8 (50.0%) children with HAs, with no statistically significant difference. Closed-set speech perception was achieved by 14/16 (87.5%) children with CI and 6/8 (75.0%) children with HAs. Language outcomes were also broadly similar between treatment groups. Some children who showed inadequate progress with HAs subsequently achieved useful open-set speech perception after CI. Current evidence does not establish a clear difference in auditory or communication outcomes between CI and HAs in children with ANSD. Treatment decisions should be individualized according to auditory development, response to amplification, speech perception, language development, and other relevant clinical factors. Further prospective comparative studies using standardized outcome measures are needed.

## Introduction and background

Auditory neuropathy spectrum disorder (ANSD) is a heterogeneous hearing disorder characterized by impaired neural synchrony despite preserved cochlear outer hair cell function. The diagnosis is typically supported by the presence of otoacoustic emissions and/or a cochlear microphonic in association with absent or markedly abnormal auditory brainstem responses (ABRs) [1-6]. ANSD may result from dysfunction at different sites along the auditory pathway, including presynaptic inner hair cell or ribbon-synapse abnormalities, auditory nerve dysfunction, and other neural transmission abnormalities. Genetic and non-genetic etiologies have both been described, contributing to substantial clinical heterogeneity [7,8].

The clinical presentation of ANSD is variable. Children may have hearing thresholds ranging from near normal to profound hearing loss, and behavioral thresholds do not necessarily predict functional listening ability. In particular, the degree of auditory neural dyssynchrony may result in speech perception that is disproportionately poor relative to the pure-tone audiogram [1,7,9]. Consequently, assessment of children with ANSD requires consideration of functional auditory performance, speech perception, language development, and progression over time rather than reliance on audiometric thresholds alone.

Management of pediatric ANSD remains challenging because auditory and language outcomes vary considerably between children. Hearing aids are commonly used as an initial intervention when behavioral thresholds and functional assessment indicate potential benefit from amplification. However, amplification increases the intensity of acoustic signals without directly correcting abnormal neural synchrony. Some children consequently demonstrate meaningful auditory and language development with hearing aids, whereas others show limited progress despite appropriately fitted amplification [1,2,7]. For children who do not obtain sufficient benefit from hearing aids, cochlear implantation may be considered. Cochlear implants bypass dysfunctional cochlear structures and provide electrical stimulation to the auditory nerve, potentially improving the neural representation of auditory information [7].

The potential benefit of cochlear implantation in ANSD is nevertheless influenced by the underlying site and severity of neural dysfunction. Auditory nerve integrity is particularly relevant because cochlear implants depend on functional neural elements capable of receiving and transmitting electrical stimulation [7]. Genetic and etiological factors may also influence outcomes [9]. In addition, the timing of implantation and the duration of auditory deprivation may affect subsequent auditory and language development [7]. These factors make treatment selection more complex than simply comparing hearing thresholds between children.

Previous studies have reported favorable outcomes following cochlear implantation in selected children with ANSD, including improvements in speech perception and auditory performance [1,7]. However, children receiving cochlear implants are frequently those who have demonstrated inadequate progress with hearing aids. Direct comparisons between cochlear implants and hearing aids are therefore particularly vulnerable to confounding by indication: the children selected for implantation may have more severe functional auditory difficulties before treatment than children who continue with amplification. This distinction is important when interpreting apparent differences between treatment groups.

## Review

Methods

This systematic review was conducted and reported in accordance with the Preferred Reporting Items for Systematic Reviews and Meta-Analyses (PRISMA) 2020 statement [10].

Eligibility Criteria

Studies were eligible if they enrolled children younger than 18 years with auditory neuropathy spectrum disorder (ANSD), defined by present otoacoustic emissions and/or a cochlear microphonic with absent or markedly abnormal auditory brainstem responses (ABRs). Studies had to report at least one quantitative auditory or communication outcome for both a cochlear implant (CI) group and a hearing aid (HA) group within the same study. Eligible outcomes included speech perception, aided thresholds or functional gain, auditory performance, speech intelligibility, auditory integration, and standardized language or developmental measures. Randomized and non-randomized comparative studies were eligible. We excluded reviews, editorials, single-patient case reports, animal studies, and studies reporting only a CI or HA group, although we used reviews for backward citation searching. Eligibility criteria were structured according to the Population, Intervention, Comparator, Outcomes, and Study design (PICOS) framework [11]. No language or date restrictions were applied (Table 1).

**Table 1:** Eligibility criteria according to the PICOS framework PICOS: Population, Intervention, Comparator, Outcomes, and Study design; ANSD: auditory neuropathy spectrum disorder; CI: cochlear implant/cochlear implantation; HA: hearing aid/hearing aids; ABR: auditory brainstem response

PICOS element	Eligibility criteria
Population (P)	Children younger than 18 years with auditory neuropathy spectrum disorder (ANSD), defined by the presence of otoacoustic emissions and/or a cochlear microphonic with absent or markedly abnormal auditory brainstem responses (ABRs).
Intervention (I)	Cochlear implantation (CI) in children with ANSD.
Comparator (C)	Hearing aids (HAs) used for auditory amplification in children with ANSD.
Outcomes (O)	At least one quantitative auditory or communication outcome, including speech perception, aided thresholds or functional gain, auditory performance, speech intelligibility, auditory integration, or standardized language/developmental measures.
Study design (S)	Randomized or non-randomized comparative studies reporting quantitative outcomes for both CI and HA groups within the same study. Reviews, editorials, single-patient case reports, animal studies, and studies reporting only a CI or HA group were excluded. No language or date restrictions were applied.

Information Sources

PubMed/MEDLINE, including PubMed Central (PMC), Web of Science Core Collection, Scopus, and the Cochrane Central Register of Controlled Trials (CENTRAL), were searched on 6 August 2026. The searches yielded 293, 1,265, 876, and 72 records, respectively, for a total of 2,506 records. Reference lists of included studies and relevant reviews were also screened. Database-specific strategies combined controlled vocabulary and free-text terms for ANSD, auditory dyssynchrony, cochlear implantation, hearing aids, and childhood. Strategies for Embase and trial registries are also provided for future updates.

Selection Process

Records were exported and de-duplicated using title, PubMed identifier (PMID), and digital object identifier (DOI). After removal of 376 duplicates, 2,130 unique records remained. Two authors (RTA and AIS) independently screened titles and abstracts and assessed potentially eligible full texts against the predefined comparative eligibility criteria. Data extraction and risk-of-bias assessment were also performed independently by two authors (MAA and AMA). Disagreements were resolved by discussion or adjudication by a third investigator (HMMR).

Data Extraction

Two authors (RTA and AIS) independently extracted study design, country, sample size, participant age, ANSD diagnostic criteria, group sizes, outcome measures, and per-group outcome data, including means and standard deviations, medians and ranges, or event counts, as reported. Discrepancies were resolved by a third investigator (HMMR). No summary statistics were imputed when outcomes were reported only as figures, medians, ranges, or proportions.

Risk-of-Bias Assessment

Risk of bias in non-randomized comparative studies was assessed independently by two authors using Risk of Bias in Non-randomized Studies of Interventions (ROBINS-I) [12]. The seven domains were confounding, selection of participants, classification of interventions, deviations from intended interventions, missing data, measurement of outcomes, and selection of the reported result. Each study received an overall judgment of low, moderate, serious, or critical risk of bias. Disagreements were resolved by a third investigator.

Synthesis Methods

Because outcomes were reported in incompatible formats, quantitative meta-analysis was not considered appropriate. Findings were therefore synthesized narratively using structured vote-counting across outcome domains, including speech perception, language, and speech production. One outcome, the proportion of children achieving at least 60% open-set speech perception, was reported using comparable per-group event counts and was expressed as a risk ratio and odds ratio with 95% confidence intervals. Because only one study contributed extractable data for this outcome, these estimates represent a single-study comparison rather than a pooled meta-analysis. The certainty of the evidence was interpreted cautiously because of the small number of studies, non-randomized designs, and substantial risk of bias. The certainty of the evidence was assessed using the GRADE (Grading of Recommendations Assessment, Development and Evaluation) approach [13].

Results

Study Selection

The four database searches conducted on 6 August 2026 identified 2,506 records: 293 from PubMed/MEDLINE, 1,265 from Web of Science, 876 from Scopus, and 72 from the Cochrane Central Register of Controlled Trials (CENTRAL). After removal of 376 duplicates, 2,130 records were screened, of which 2,090 were excluded. Of 40 reports sought for retrieval, 2 eligible reports could not be retrieved, leaving 38 reports for full-text assessment. Thirty-two reports were excluded because they lacked a head-to-head cochlear implant (CI) versus hearing aid (HA) comparison or extractable per-arm data (n = 28), or because the auditory neuropathy spectrum disorder (ANSD) subgroup could not be separated from a mixed population (n = 4). Five studies, reported in six publications, were included [1-5]. Two publications reported different outcomes from the same Melbourne cohort and were therefore treated as a single study (Figure 1) [1,2].

**Figure 1:**
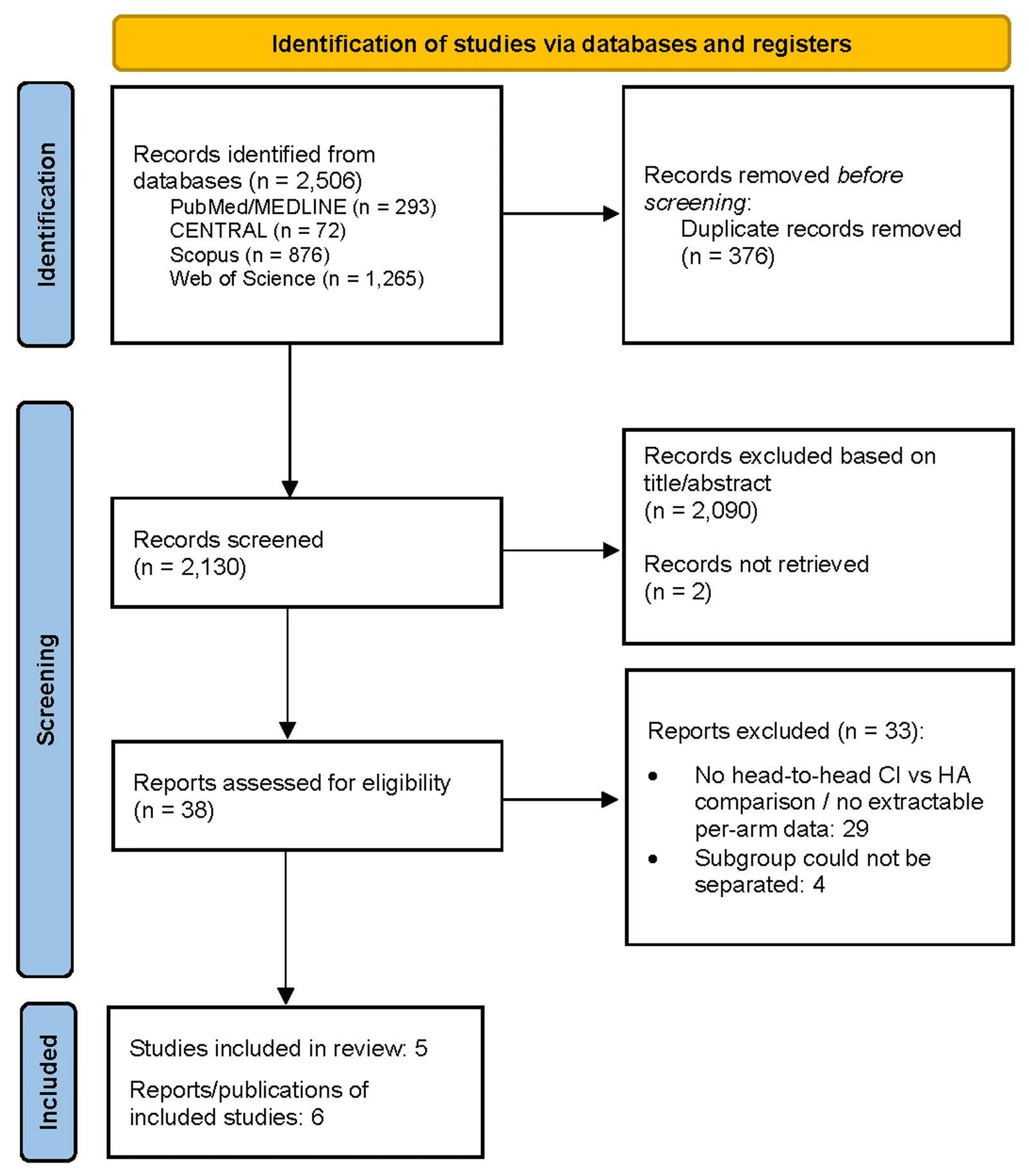
PRISMA flow diagram depicting the study selection process for the systematic review Following the identification of records through database searching and other sources, duplicates were removed, and the remaining records were screened. Full-text articles were assessed for eligibility, with exclusions documented along with reasons. Studies meeting the inclusion criteria were included in the final synthesis. PRISMA: Preferred Reporting Items for Systematic Reviews and Meta-Analyses

Study Characteristics

The included studies comprised prospective and retrospective cohorts from Australia, the United States, and Germany, as well as one chart review from Saudi Arabia [1-6]. CI groups included 10-19 children, and HA groups included 8-27 children [1,3,4]. The two publications reporting the Melbourne cohort provided different outcomes and were treated as a single data source, with participants counted once (Tables 2-3) [1,2].

**Table 2:** Characteristics of included studies This table provides the characteristics of the five included studies (six reports) comparing CI and HA in children with ANSD. The table summarizes study location and design, sample sizes, participant characteristics, and reported outcome measures. Rance [1] and Rance [2] represent the same Melbourne cohort and were treated as a single data source; participants were not double-counted. Almishaal [6] was included in the systematic review but excluded from the quantitative synthesis because of its prevalence-study design, aided-threshold outcome, and lack of per-arm sample sizes and standard deviations. Two additional eligible reports could not be retrieved because full texts were unavailable. CI: cochlear implants; HA: hearing aids; ANSD: auditory neuropathy spectrum disorder; NR: not reported; CNC: consonant–nucleus–consonant; PPC: percentage-phonemes-correct; PPVT: Peabody Picture Vocabulary Test; IT-MAIS:, Infant–Toddler Meaningful Auditory Integration Scale; HSM: Hochmair–Schulz–Moser; PTA: pure-tone average

Study	Country	Design	CI, n	HA, n	Population	Outcome measures
Rance [1]	Australia	Prospective cohort	10	10	Children with auditory neuropathy/auditory dys-synchrony; test age approximately 90 months	Consonant–nucleus–consonant (CNC) open-set phoneme score
Rance [2]	Australia	Cohort (same cohort as Rance [1])	10	10	School-aged children with auditory neuropathy/auditory dys-synchrony	Language quotient; percentage-phonemes-correct (PPC); Peabody Picture Vocabulary Test (PPVT)
Pelosi [3]	United States	Retrospective cohort	16	8	Children with auditory neuropathy spectrum disorder (ANSD)	Infant–Toddler Meaningful Auditory Integration Scale (IT-MAIS); open- and closed-set speech perception
Ehrmann-Müller [4]	Germany	Retrospective cohort	17	8	Children with ANSD	Functional gain (dB); monosyllabic speech score (%); Hochmair–Schulz–Moser (HSM) test
Ching [5]	Australia	Population cohort	19	27	Children with ANSD (n = 47), age 3 years	Global development/language (factor z-score)
Almishaal [6]	Saudi Arabia	Retrospective chart review	NR	NR	ANSD (101 of 1,025 children with sensorineural hearing loss)	Aided pure-tone average (PTA) / speech-detection threshold (dB)

**Table 3:** Per-arm outcome data extracted from retrieved full texts Per-arm auditory, language, and speech outcomes reported in the retrieved full-text studies comparing CI with HA in children with ANSD. Values are presented exactly as reported in the source studies; no values were imputed. Unless otherwise specified, continuous data are presented as mean (standard deviation (SD)). Median values, ranges, percentages, event counts, and graphically reported results are identified accordingly. Quantitative synthesis indicates whether the reported data were considered suitable for effect-size calculation. Rance [1] and Rance [2] report the same cohort and were treated as one data source; participants were not double-counted. The Ehrmann-Müller [4] device comparison was performed in a subset of 6 CI and 6 HA participants and was reported graphically. Almishaal [6] was excluded from the quantitative synthesis because only a P value was reported. Ching [5] assessed device type as a covariate in an adjusted analysis rather than reporting per-arm outcome estimates. CI: cochlear implants; HA: hearing aids; ANSD: auditory neuropathy spectrum disorder; CNC: consonant–nucleus–consonant; CI: cochlear implant; HA: hearing aid; IT-MAIS: Infant–Toddler Meaningful Auditory Integration Scale; NR: not reported; PPC: percentage-phonemes-correct; PPVT: Peabody Picture Vocabulary Test; PTA: pure-tone average; SD: standard deviation; SDT: speech-detection threshold

Study	Outcome	Timepoint	CI, n	CI outcome	HA, n	HA outcome	Quantitative synthesis
Rance [1]	Open-set speech perception, CNC phoneme score (%)	Post-treatment, ~90 months	10	59.6 (20.6)	10	55.1 (24.8)	Yes; standardized mean difference
Rance [2]	Language quotient	School age	10	0.68 (0.08)	10	0.76 (0.21)	Yes; standardized mean difference
Rance [2]	Speech production, PPC deviation from age-expected value (%)	School age	10	14.8 (11.0)	10	9.3 (6.4)	Yes; standardized mean difference
Rance [2]	Receptive vocabulary, PPVT-derived (%)	School age	10	80.9 (11.1)	10	88.1 (7.6)	Uncertain; metric requires confirmation
Pelosi [3]	Open-set speech perception ≥60%	Post-treatment	16	6/16 (37.5%)	8	4/8 (50.0%)	Yes; dichotomous
Pelosi [3]	Closed-set speech perception achieved	Post-treatment	16	14/16 (88%)	8	6/8 (75%)	Yes; dichotomous
Pelosi [3]	IT-MAIS score	Pre-treatment	16	Median 13	8	Median 30	No; median only, SD not reported
Ehrmann-Müller [4]	Functional gain (dB)	Aided	17	32–50	8	32–65	No; range only
Ehrmann-Müller [4]	Open-set monosyllabic speech perception in quiet (%)	Aided	6	Figure; non-significant Mann–Whitney U test; CI tended higher	6	Figure; non-significant Mann–Whitney U test	No; figure only, no ≥60% per-arm counts
Almishaal [6]	Aided SDT/PTA (dB)	Aided	NR	Improved versus HA, P < 0.001	NR	Reference	No; P value only
Ching [5]	Global development/language, factor z-score	Age 3 years	19	Device type was not a significant adjusted predictor; per-arm outcome not reported	27	—	No; no per-arm mean/SD or counts

Risk of Bias

Four of five studies had serious overall risk of bias when assessed using Risk of Bias In Non-randomized Studies of Interventions (ROBINS-I), primarily because treatment assignment was influenced by clinical response to amplification. Thus, children receiving CI were generally those who had failed to progress with HAs [1,3,4]. One study had a moderate risk of bias because of population-based sampling and adjustment for potential confounders [5]. Additional concerns included missing outcome data and selective reporting in individual studies (Table 4) [4,6].

**Table 4:** Risk of bias of included comparative studies Risk-of-bias judgments for the five included comparative studies, assessed using the Risk Of Bias In Non-randomized Studies of Interventions (ROBINS-I) tool. The table presents judgments across seven domains: D1, bias due to confounding; D2, bias in selection of participants into the study; D3, bias in classification of interventions; D4, bias due to deviations from intended interventions; D5, bias due to missing data; D6, bias in measurement of outcomes; and D7, bias in selection of the reported result. Overall judgments are based on the domain-level assessments. Rance [1,2] represents the same Melbourne cohort reported in two publications and was treated as a single study. Almishaal [6] was included in the review but excluded from the quantitative synthesis.

Study	D1: Confounding	D2: Selection of participants	D3: Classification of interventions	D4: Deviations from intended interventions	D5: Missing data	D6: Measurement of outcomes	D7: Selection of reported results	Overall
Rance [1,2]	Serious	Moderate	Low	Low	Low	Moderate	Low	Serious
Pelosi [3]	Serious	Moderate	Low	Moderate	Moderate	Moderate	Moderate	Serious
Ehrmann-Müller [4]	Serious	Moderate	Low	Moderate	Serious	Moderate	Moderate	Serious
Ching [5]	Moderate	Low	Low	Low	Moderate	Low	Moderate	Moderate
Almishaal [6]	Serious	Moderate	Low	Moderate	Serious	Moderate	Serious	Serious

Synthesis of Findings

For speech perception, one study reported mean open-set consonant-nucleus-consonant (CNC) phoneme scores of 59.6% (standard deviation (SD), 20.6) with CI and 55.1% (SD, 24.8) with HAs, with substantial overlap between groups [1]. Another study reported open-set speech perception of at least 60% in 6 of 16 children (37.5%) with CI and 4 of 8 children (50.0%) with HAs; closed-set speech perception was achieved by 14 of 16 children (87.5%) and 6 of 8 children (75.0%), respectively [3]. The corresponding risk ratio was 0.75 (95% confidence interval (CI), 0.29-1.92), and the odds ratio was 0.60 (95% CI, 0.11-3.34) [3]. Both estimates were based on a single study and were not statistically significant. A third study reported open-set monosyllabic scores ranging from 35% to 100% with either device and found no significant difference in a matched subgroup [4].

Language outcomes were also similar between devices. The Melbourne cohort reported comparable language quotients for children using CI and HAs, with no significant group difference [2]. In a population-based study, device type did not significantly predict global developmental or language outcomes at 3 years after adjusting for potential confounders [5].

For speech production, the Melbourne cohort reported percentage-phonemes-correct deviations from age-expected values of 14.8% (SD, 11.0) with CI and 9.3% (SD, 6.4) with HAs; the difference was not statistically tested, and the smaller HA group was numerically closer to age norms [2].

One chart review reported significantly greater improvements in aided pure-tone and speech-detection thresholds with CI than with HAs (P < 0.001) [6]. Because per-group sample sizes and variability measures were not reported, these findings were not included in the quantitative synthesis [6].

Overall, no included study demonstrated a statistically significant difference between CI and HAs in speech perception or language outcomes [1-5]. The available evidence suggests that some children progress adequately with amplification, whereas children who fail to progress with HAs may achieve useful open-set speech perception after CI [1,3,4]. Because outcomes were reported using heterogeneous measures and formats [1-6], a pooled meta-analysis was not appropriate.

Discussion

Significance of Findings

The findings of this review are broadly consistent with the existing literature, indicating substantial variability in auditory outcomes among children with ANSD and suggesting that treatment response cannot be predicted reliably from audiometric thresholds alone [1-7]. Previous studies have demonstrated that some children achieve meaningful speech and language development with HAs, whereas others with inadequate progress may obtain useful auditory performance following CI [1-4]. The absence of a statistically significant difference between CI and HAs in the comparative studies included in this review should therefore be interpreted in the context of the heterogeneous nature of ANSD and the clinical factors influencing device selection. In particular, auditory nerve integrity, underlying etiology, response to amplification, timing of implantation, and duration of auditory deprivation may contribute to differences in outcomes between individual children [7]. Thus, the findings reinforce the current literature supporting an individualized approach to management, with longitudinal assessment of functional auditory performance, speech perception, and language development informing decisions regarding continued amplification or cochlear implantation [1-7].

Implications for Clinical Practice

The findings support an individualized approach to device selection in children with ANSD. Hearing aids remain appropriate for children who demonstrate meaningful auditory progress with amplification, while cochlear implantation may be considered for children who show inadequate auditory, speech, or language development despite appropriately fitted hearing aids [1,2,7]. Clinical decision-making should incorporate functional auditory performance rather than audiometric thresholds alone because the relationship between thresholds and functional outcomes in ANSD can be poor [7,8].

Assessment of cochlear nerve integrity and consideration of the underlying etiology are also important when evaluating candidacy for cochlear implantation [7-9]. The evidence does not support using the diagnosis of ANSD alone as an indication for either cochlear implantation or continued hearing-aid use. Instead, treatment should be guided by longitudinal auditory development, response to amplification, speech perception, language development, and other patient-specific factors.

Strengths and Limitations of the Present Review

This review has several strengths. It specifically addressed the clinically important question of cochlear implantation versus hearing aids in children with ANSD and restricted inclusion to comparative studies reporting outcomes for both treatment groups. The review also used predefined eligibility criteria, independent study selection and data extraction, and formal risk-of-bias assessment using ROBINS-I. The systematic search incorporated four major bibliographic databases and included backward citation searching.

Several limitations should nevertheless be acknowledged. The number of eligible studies was small, and two eligible reports could not be retrieved. All included comparative studies were non-randomized, and most had a serious risk of bias. Outcome measures and reporting formats varied considerably, preventing a pooled meta-analysis. Some studies did not provide sufficient per-arm data for quantitative synthesis, while one included study was a population-based analysis in which device type was considered as a covariate rather than the primary comparative intervention. These limitations reduce the certainty and generalizability of the findings.

## Conclusions

Current evidence does not establish a clear difference in auditory or communication outcomes between cochlear implants and hearing aids in children with auditory neuropathy spectrum disorder. The available evidence is limited by the small number of comparative studies, non-randomized designs, heterogeneity of outcome measures, and risk of bias. Treatment decisions should therefore be individualized based on longitudinal auditory development, response to appropriately fitted amplification, speech perception, language development, and other relevant clinical factors. Further prospective comparative studies using standardized outcome measures are needed to better inform device selection in this population.

## References

[1] Rance G, Barker EJ (2008). Speech perception in children with auditory neuropathy/dyssynchrony managed with either hearing AIDS or cochlear implants. Otol Neurotol.

[2] Rance G, Barker EJ (2009). Speech and language outcomes in children with auditory neuropathy/dys-synchrony managed with either cochlear implants or hearing aids. Int J Audiol.

[3] Pelosi S, Wanna G, Hayes C (2013). Cochlear implantation versus hearing amplification in patients with auditory neuropathy spectrum disorder. Otolaryngol Head Neck Surg.

[4] Ehrmann-Müller D, Cebulla M, Rak K, Scheich M, Back D, Hagen R, Shehata-Dieler W (2019). Evaluation and therapy outcome in children with auditory neuropathy spectrum disorder (ANSD). Int J Pediatr Otorhinolaryngol.

[5] Ching TY, Day J, Dillon H (2013). Impact of the presence of auditory neuropathy spectrum disorder (ANSD) on outcomes of children at three years of age. Int J Audiol.

[6] Almishaal AA, Saleh S, Alferaih H, Alhelo O (2022). Prevalence, risk factors, and audiological characteristics of auditory neuropathy. Int J Audiol.

[7] Harrison RV, Gordon KA, Papsin BC, Negandhi J, James AL (2015). Auditory neuropathy spectrum disorder (ANSD) and cochlear implantation. Int J Pediatr Otorhinolaryngol.

[8] Kaga K (2016). Auditory nerve disease and auditory neuropathy spectrum disorders. Auris Nasus Larynx.

[9] Sun L, Lin Z, Zhang J, Shen J, Wang X, Yang J (2022). Genetic etiological analysis of auditory neuropathy spectrum disorder by next-generation sequencing. Front Neurol.

[10] Page MJ, McKenzie JE, Bossuyt PM (2021). The PRISMA 2020 statement: an updated guideline for reporting systematic reviews. Rev Esp Cardiol (Engl Ed).

[11] Schardt C, Adams MB, Owens T, Keitz S, Fontelo P (2007). Utilization of the PICO framework to improve searching PubMed for clinical questions. BMC Med Inform Decis Mak.

[12] Sterne JA, Hernán MA, Reeves BC (2016). ROBINS-I: a tool for assessing risk of bias in non-randomised studies of interventions. BMJ.

[13] Guyatt GH, Oxman AD, Vist GE, Kunz R, Falck-Ytter Y, Alonso-Coello P, Schünemann HJ (2008). GRADE: an emerging consensus on rating quality of evidence and strength of recommendations. BMJ.

